# Automated detection of nocturnal motor seizures using an audio‐video system

**DOI:** 10.1002/brb3.2737

**Published:** 2022-08-08

**Authors:** Sidsel Armand Larsen, Daniella Terney, Tim Østerkjerhuus, Torsten Vinding Merinder, Kaapo Annala, Andrew Knight, Sándor Beniczky

**Affiliations:** ^1^ Department of Clinical Neurophysiology Danish Epilepsy Centre Dianalund Denmark; ^2^ Department of Clinical Neurophysiology Aarhus University Hospital Aarhus Denmark; ^3^ Neuro Event Labs Tampere Finland; ^4^ Department of Clinical Medicine Aarhus University Aarhus Denmark

**Keywords:** audio‐video surveillance system, automated seizure detection, hypermotor seizures, nocturnal seizures, tonic‐clonic seizures

## Abstract

**Background:**

Unsupervised nocturnal tonic‐clonic seizures (TCSs) may lead to sudden unexpected death in epilepsy (SUDEP). Major motor seizures (TCSs and hypermotor seizures) may lead to injuries. Our goal was to develop and validate an automated audio‐video system for the real‐time detection of major nocturnal motor seizures.

**Methods:**

In this Phase‐3 clinical validation study, we assessed the performance of automated detection of nocturnal motor seizures using audio‐video streaming, computer vision and an artificial intelligence‐based algorithm (Nelli). The detection threshold was predefined, the validation dataset was independent from the training dataset, patients were prospectively recruited, and the analysis was performed in real time. The gold standard was based on expert evaluation of long‐term video electroencephalography (EEG). The primary outcome was the detection of nocturnal major motor seizures (TCSs and hypermotor seizures). The secondary outcome was the detection of other (minor) nocturnal motor seizures.

**Results:**

We recruited 191 participants aged 1–72 years (median: 20 years), and we monitored them for 4183 h during the night. Device deficiency was present 10.5% of the time. Fifty‐one patients had nocturnal motor seizures during the recording. The sensitivity for the major motor seizures was 93.7% (95% confidence interval: 69.8%–99.8%). The system detected all 11 TCS and four out of five (80%) hypermotor seizures. For the minor motor seizure types, the sensitivity was low (8.3%). The false detection rate was 0.16 per h.

**Conclusion:**

The Nelli system detects nocturnal major motor seizures with a high sensitivity and is suitable for implementation in institutions (hospitals, residential care facilities), where rapid interventions triggered by alarms can potentially reduce the risk of SUDEP and injuries.

## 1 INTRODUCTION

Nocturnal generalized tonic‐clonic seizures (TCSs), including focal‐to‐bilateral TCSs (formerly known as secondarily generalized TCSs), represent the major risk factor for sudden unexpected death in epilepsy (SUDEP) patients (Devinsky et al., 2016). Patients without nocturnal surveillance, not sharing a bedroom and experiencing TCS, have a 67‐fold increase in the risk of SUDEP (Sveinsson et al., 2020). Postictal interventions, such as stimulation, repositioning, or clearing the airways of the patient, may be protective against SUDEP (Surges et al., 2009). Major convulsive seizures, such as TCS and fulminant hypermotor seizures, may lead to injuries (Salas‐Puig et al., 2019). Most nocturnal seizures (85%) remain unreported by the patients (Hoppe et al., 2007).

A recently published clinical practice guideline of the International League Against Epilepsy (ILAE) and the International Federation of Clinical Neurophysiology (IFCN) recommends the use of clinically validated wearable devices for the automated detection of TCS in unsupervised patients, where alarms can result in rapid intervention (Beniczky et al., 2021a, 2021b). However, the currently available automated seizure detection devices, validated in Phase 3 clinical trials, are all obtrusive (Beniczky et al., 2021a, 2021b). Our goal was to develop and validate a nonobtrusive, contactless automated seizure detection system based on audio‐video signals for use in institutions (hospitals, residential care facilities) where personnel are available for rapid intervention during the night, but the number of patients exceeds the possibility of continuous, human/visual surveillance (which is the case in most institutions).

We conducted a Phase‐3 clinical validation study (Beniczky & Ryvlin, 2018): We used a predefined algorithm and detection thresholds for real‐time detection of nocturnal major motor seizures in a prospective, multicenter study. The primary outcome was the accuracy of detecting major convulsive seizures (TCS and hypermotor seizures). The secondary outcome was the detection of other nocturnal motor seizures.

## 2 METHODS

Consecutive patients were prospectively recruited at the Danish Epilepsy Centre and at Aarhus University Hospital between October 14, 2019, and June 18, 2021. The study was approved by the regional ethics committee (SJ‐756). All patients or parents/guardians of patients gave their written, informed consent prior to the study. Inclusion criteria were (1) admission to noninvasive inpatient long‐term video‐EEG monitoring in the epilepsy monitoring unit (EMU) and (2) patients having nocturnal motor seizures. We excluded from the analysis of sensitivity (1) patients who did not have any nocturnal motor seizures during the monitoring, (2) patients with unclassified seizures (when the gold standard could not provide a seizure classification), and (3) patients with failed recordings by the seizure detection system (device deficiency). All recruited patients and the entire monitoring time (without any exclusions) were used to determine the percentage of device deficiency time and the false alarm rate.

For nonobtrusive automated seizure detection, we used an audio‐video‐based system (Nelli) approved by the European Union (CE‐mark) consisting of a specialized, high‐definition camera, and microphone (Ojanen et al., 2021). The signals were streamed online to a central computer using a secure internet connection. Data were processed in real time using a previously developed artificial intelligence‐based algorithm and a predefined seizure detection threshold for major motor seizures (TCS and hypermotor seizures). Nelli used computer vision and machine learning to detect seizure events (Supplementary Material 1). Data analysis was automated and blinded to any other data.

The gold standard for identifying seizures was a clinical expert evaluation of long‐term video‐electroencephalography (EEG) recordings blinded to the automated detection. The recording array comprised EEG electrodes (19–25 electrodes for diagnostic monitoring and 40 electrodes for patients undergoing presurgical evaluation), electrocardiography (ECG) and surface electromyography electrodes, placed as specified in the guidelines of the IFCN (Seeck et al., 2017). Two experts (SAL and DT) independently evaluated the video‐EEG recording data and classified the recorded clinical episodes. Disagreements were resolved by consensus discussions involving a third expert (SB). Automated detection was then compared with the gold standard.

We conducted the study and reported the results according to the proposed standards for testing and clinical validation of seizure detection devices (Beniczky & Ryvlin, 2018). The primary outcome was the detection of major convulsive seizures (TCS and hypermotor seizures). The secondary outcome was the detection of other nocturnal motor seizures. We determined the sensitivity, false alarm rate, and device deficiency time.

## 3 RESULTS

We recruited 191 participants with known or suspected epilepsy (98 female; age: 10 months to 72 years, median: 20 years) and monitored them during the night for a total of 4183 h. The device deficiency was 10.5% (441 h without streamed data, 3742 h with streamed and analyzed data).

**FIGURE 1:**
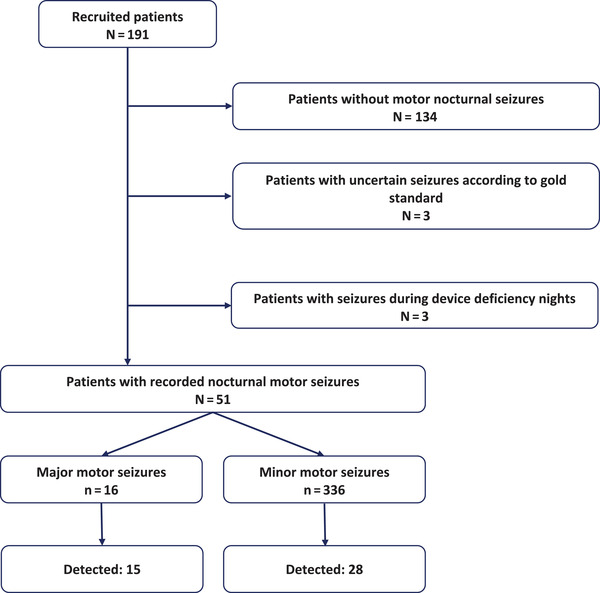
Study flow diagram: From recruited patients to the analyzed seizures

During device deficiency periods, three patients had a total of 10 nocturnal motor seizures. One hundred thirty‐four patients did not have nocturnal motor seizures in the EMU, and three patients had uncertain/unclassified seizures according to the gold standard. These patients were excluded from the sensitivity analysis (Figure 1).

**FIGURE 2:**
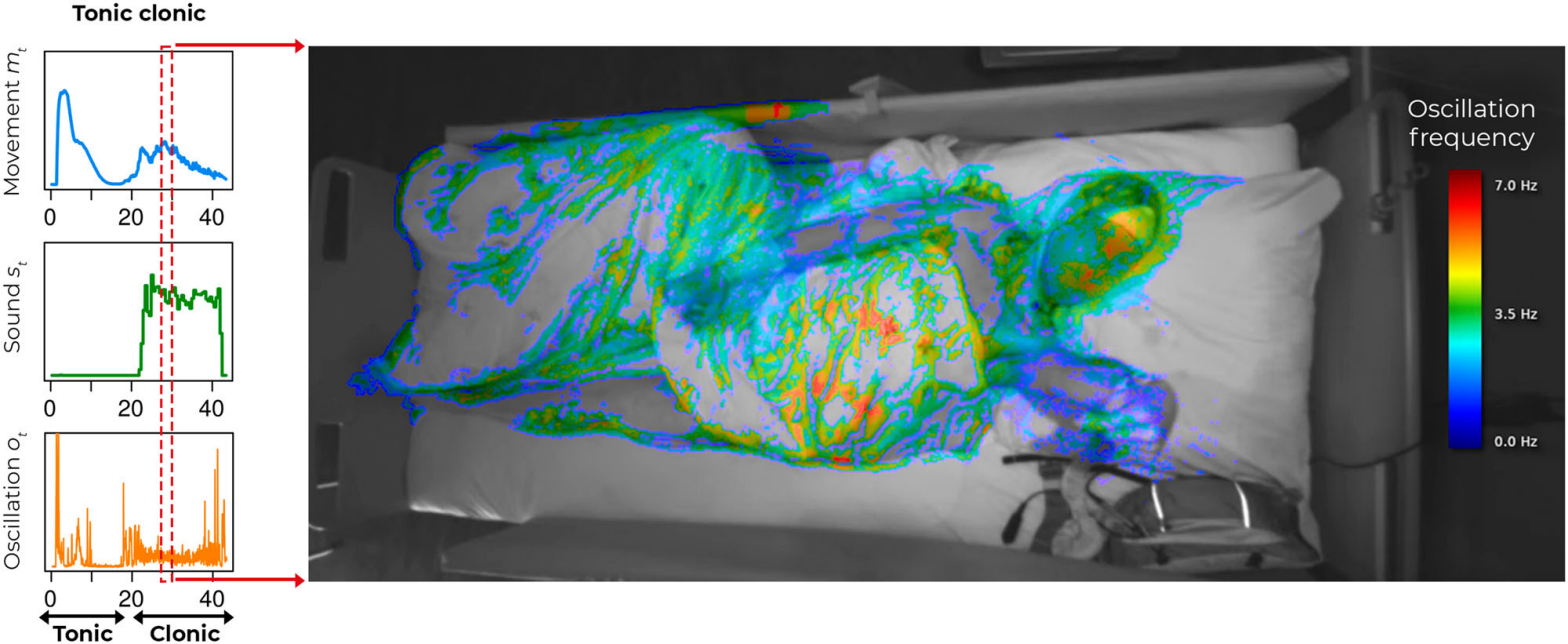
Example of the algorithm output during a generalised tonic‐clonic seizure. Computer‐vision features are superimposed on the body parts where they are active in the clonic phase of the seizure

Fifty‐one patients (32 males; age: 2–72 years; median: 16 years) had nocturnal motor seizures during the streamed recording hours. Figure 2 shows the automated features extracted during computer vision and seizure detection. Of the 16 major convulsive seizures, Nelli detected 15 seizures (sensitivity: 93.7%; 95% confidence interval: 69.8%–99.8%). All TCSs (11 seizures from 10 patients) were detected (sensitivity: 100%; 95% CI: 71.5%–100%). Four out of five hypermotor seizures were detected (sensitivity: 80%; 95% CI: 28.4%–99.5%). Of the 336 minor motor seizures, Nelli detected 28 (sensitivity: 8.3%; 95% CI: 5.6%–11.8%; table in Supplementary Material 2).

The Nelli system had 581 false alarms in 181 patients (3742 h). The false alarm rate was 0.16 per h (one false alarm in 6 h and 30 min, on average). The median rate per patient was 0.07 per h (interquartile range: 0–0.19). Sixty‐three percent of all patients had at least one false alarm. A total of 333/581 false alarms were triggered by physiological movements in sleep: stretching, scratching, and turning in bed; 231/581 were triggered by other persons in view: nurse, technical staff, or parent; 17/581 false detections were nonepileptic paroxysmal events.

## 4 DISCUSSION

In this Phase‐3, prospective, multicenter, validation study, we found that automated real‐time audio‐video analysis using the Nelli system had high sensitivity (93.7%) for detecting nocturnal major motor seizures. The system detected all 11 TCSs, which is of utmost importance because unsupervised nocturnal TCSs are the major cause of SUDEP (Ryvlin et al., 2013), and the recently published ILAE–IFCN clinical practice guideline recommends the use of clinically validated devices for the automated detection of TCS in unsupervised patients, where alarms can result in rapid intervention (Beniczky et al., 2021a, 2021b). Nelli detected four out of five (80%) nocturnal hypermotor seizures, but the sensitivity for the other (minor) nocturnal motor seizures was too low for clinical implementation (8.3%).

The sensitivity of the Nelli system for TCS is similar or slightly superior to the wearable seizure detection devices that have been validated in Phase 3 studies (Beniczky et al., 2021a, 2021b). The advantage of the Nelli system, compared to these systems is that it is nonobtrusive. The disadvantage is that the current version of the system is limited to the area under the fixed camera view and nocturnal surveillance as tested in this study. Nevertheless, nocturnal seizure detection is most important for preventing SUDEP. Previous Phase‐2, retrospective, single‐center studies on seizure detection using automated video analysis reported similar sensitivity to our phase‐3 study (Geertsema et al., 2018; van Westrhenen et al., 2021, 2020). The sensitivity was 78%–100% for TCS and 60%–73% for hypermotor seizures (Geertsema et al., 2018; van Westrhenen et al., 2021, 2020). The number of patients with seizures in these retrospectives, Phase‐2 studies was comparable to our study (12 patients in residential care; Geertsema et al., 2018); six children with convulsive seizures and two children with hypermotor seizures (van Westrhenen et al., 2021, 2020). However, the number of recorded seizures was much higher (50 convulsive seizures in the residential care (Geertsema et al., 2018); 69 convulsive seizures and 161 hypermotor seizures in the pediatric series (van Westrhenen et al., 2021, 2020), compared to our study. This is due to the longer video monitoring time at home and in residential care facilities. However, that was at the cost of the gold standard: EEG was not available, and not the entire (continuous) monitored period was assessed by physicians (Geertsema et al., 2018; van Westrhenen et al., 2021, 2020). We opted to conduct the study in the EMU, where a robust gold standard was available. However, that resulted in a lower number of seizures per patient. The number of false alarms in the retrospective, Phase‐2 studies was relatively low: 0.05–0.78 per night (Geertsema et al., 2018; van Westrhenen et al., 2021, 2020).

The false alarm rate of 0.16 per h (one false alarm in 6.5 h) is too high for a setting where automated detections send alarms to parents or carers who sleep during the night. However, in an institutional setting (hospitals, residential care facilities) where personnel are present during the night shift, implementation of the real‐time seizure alarm using the Nelli system seems to be feasible and would significantly decrease the burden of continuous video surveillance during the night.

A major limitation of the current version of the Nelli system for this use‐case scenario is the high percentage of device deficiency time (10.5%). These were caused by unstable secure internet connections. Nelli service performs daily checks of the devices to reduce deficiency time. In the future, deficiency may be improved by using two internet connections in parallel from two different providers. In spite of the high number of recruited patients, the relatively low number in the subgroup of patients with nocturnal major motor seizures is a limitation in our study. Only 51 patients had nocturnal motor seizures, and 10 of them had major motor seizures during the nocturnal recordings. In EMU, the occurrence of generalized TCS is considered an unwanted side‐effect (unless that is the only seizure type of the patients). This explains why we were not able to record more seizures in this study conducted in the EMU.

In conclusion, this Phase‐3 validation study showed that automated real‐time seizure detection using audio‐video analysis has a high sensitivity for TCS and is suitable for nocturnal surveillance of patients in institutions (hospitals, residential care facilities) where personnel are present during the night, allowing rapid intervention.

## AUTHOR CONTRIBUTIONS

*Conceptualization, formal analysis, writing–original draft*: Sidsel Armand Larsen. *Formal analysis, writing–review and editing*: Daniella Terney. *Formal analysis, writing–review and editing*: Tim Østerkjerhuus. *Conceptualization, writing–review and editing*: Torsten Vinding Merinder. *Formal analysis, writing–review and editing*: Kaapo Annala. *Formal analysis, writing–review and editing*: Andrew Knight. *Conceptualization, formal analysis, writing–review and editing*: Sándor Beniczky.

## CONFLICT OF INTEREST

Kaapo Annala and Andrew Knight are shareholders and employees of the Neuro Event Labs. They extracted and summarized the results of the automated analysis. This was done blinded to all other data. The SAL institution received research funding from the Neuro Event Labs, covering compensation for SAL´s working hours for this project.

### PEER REVIEW

The peer review history for this article is available at https://publons.com/publon/10.1002/brb3.2737

## Supporting information


**Supplementary FIGURE 1** Signal processing and machine learning turn video and audio feed into biomarkers, events, and findings.

Supplementary Material

## Data Availability

The data that support the findings of this study are available in the Supplementary Material of this article.
