# Supplementary material for: Automated detection of nocturnal motor seizures using an audio‐video system
Source: Brain Behav. 2022 Aug 8;12(9):e2737. doi: 10.1002/brb3.2737 (PMC9480955; doi:10.1002/brb3.2737)
Supplement: Supplementary file 1 — Supplementary FIGURE 1 Signal processing and machine learning turn video and audio feed into biomarkers, events, and findings. [file BRB3-12-e2737-s002.pdf]

Supplementary Figure 1

Signal processing and machine learning turns video and audio feed into biomarkers, events and findings.

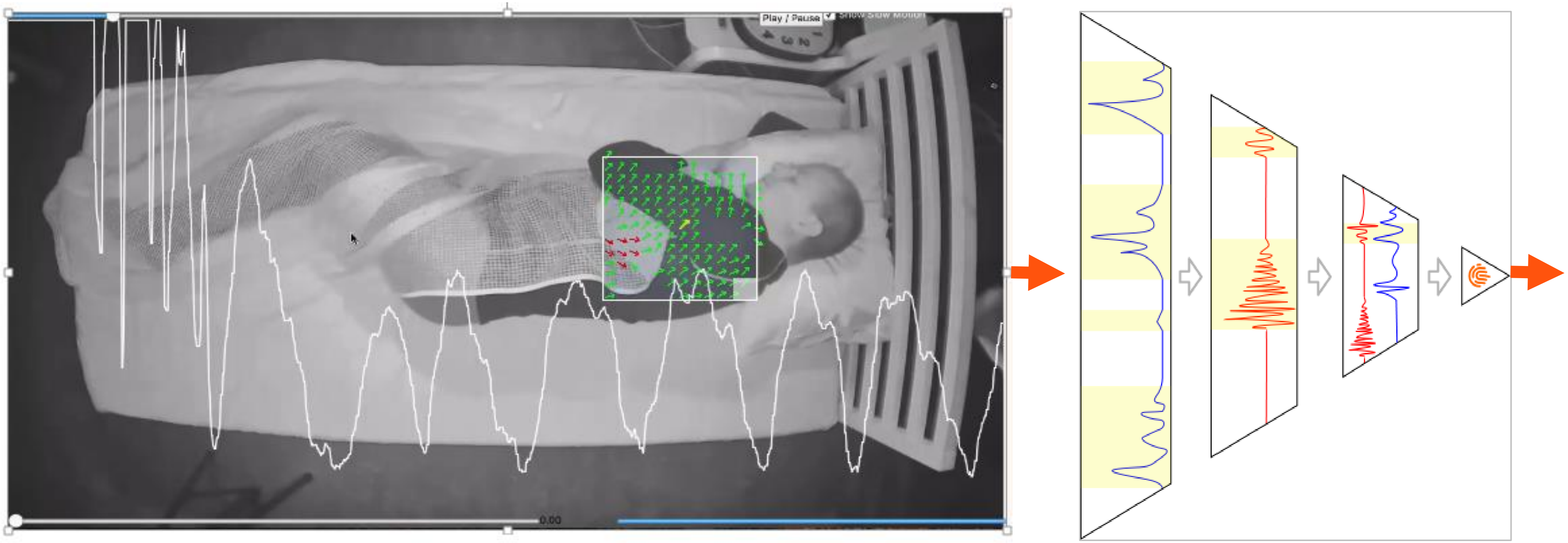

Biomarkers

- Respiration
- Clonic movement
- Seizure audio fingerprint
- Tonic posture
- Pose estimation
- Joint velocity
- Spasm
- Twitches
- Eyes open, deviation
- Lip smacking
- Hand orientation
