# Supplementary material for: Automated detection of nocturnal motor seizures using an audio‐video system
Source: Brain Behav. 2022 Aug 8;12(9):e2737. doi: 10.1002/brb3.2737 (PMC9480955; doi:10.1002/brb3.2737)
Supplement: Supplementary file 2 — Supplementary Material [file BRB3-12-e2737-s001.pdf]

Supplementary material 2

Seizure types		Total recorded seizures	Detected by Nelli	Sensitivity % (detected/total)
Major motor seizures	Tonic-clonic (Generalised and FBTCS)	11	11	100
	Hypermotor	5	4	80
Minor motor seizures	Tonic	68	9	13
	Focal motor	101	17	17
	Spasm	95	0	0
	Myoclonic	57	0	0
	Subtle seizure	15	2	13
